# Lysosomal Cathepsins in Myocardial Ischemia–Reperfusion Injury: A Druggable Nexus for Multi-Modal Cell Death

**DOI:** 10.3390/cells15201895

**Published:** 2026-10-17

**Authors:** Yilong Wang, Jianyong Du, Erdan Dong, Wentao Sun

**Affiliations:** 1 NHC Key Laboratory of Cardiopulmonary Rehabilitation and Functional Recovery, School of Health and Life Sciences, University of Health and Rehabilitation Sciences, Qingdao 266113, China; dujianyong66@uhrs.edu.cn; 2 Research Center for Cardiopulmonary Rehabilitation, University of Health and Rehabilitation Sciences Qingdao Hospital ( Qingdao Municipal Hospital), Qingdao 266071, China; donged@uhrs.edu.cn; 3 NHC Key Laboratory of Cardiopulmonary Rehabilitation and Functional Recovery, School of Medicine, University of Health and Rehabilitation Sciences, Qingdao 266113, China; 4 Department of Cardiology and Institute of Vascular Medicine, Peking University Third Hospital, Beijing 100191, China; 5 State Key Laboratory of Vascular Homeostasis and Remodeling, Peking University, Beijing 100191, China; 6 The Institute of Cardiovascular Sciences, Peking University, Beijing 100191, China; 7 Research Unit of Medical Science Research Management/Basic and Clinical Research of Metabolic Cardiovascular Diseases, Chinese Academy of Medical Sciences, Beijing 100871, China

**Keywords:** cathepsins, lysosomal membrane permeabilization, myocardial ischemia–reperfusion injury, regulated cell death, PANoptosis, ferroptosis, inflammasome

## Abstract

**Highlights:**

**What are the main findings?**

- Lysosomal membrane permeabilization releases cathepsins that synchronize apoptosis, pyroptosis, ferroptosis, and PANoptosis through one proteolytic hub in MIRI.
- Compensatory activation across death modalities explains why single-pathway cardioprotective monotherapies have yielded limited clinical benefit.

**What are the implications of the main findings?**

- The lysosomal-cathepsin axis is an upstream druggable node for simultaneous multi-target cardioprotection in ischemic heart disease.
- Death-phenotype biomarker panels could enable patient stratification and adaptive, mechanism-matched trial designs in STEMI.

**Abstract:**

Myocardial ischemia–reperfusion injury (MIRI) remains a central challenge in coronary artery disease. Timely revascularization salvages ischemic myocardium yet simultaneously triggers cardiomyocyte death that offsets therapeutic benefit. Apoptosis, necroptosis, pyroptosis, and ferroptosis are all executed during ischemia–reperfusion, yet their temporal coordination and inter-regulation remain incompletely understood. Consequently, monotherapies targeting single death pathways have repeatedly failed in clinical translation. This review examines the pharmacological evidence that cathepsin-driven lysosomal membrane permeabilization (LMP) operates as a proteolytic nexus coordinating these death programs. Cytosolic cathepsins cleave Bid to amplify mitochondrial apoptosis, bind and activate the NOD-like receptor protein 3 (NLRP3) inflammasome to drive pyroptosis, and degrade ferritin to mobilize iron for ferroptosis, while participating in PANoptotic execution. In endothelial cells, the same machinery promotes microvascular obstruction and no-reflow. We further evaluate circulating cathepsins and death-pathway biomarkers for patient stratification and appraise isoform-selective inhibitors, multi-target nanomedicine, and biomarker-guided adaptive trial designs. Positioning the lysosomal-cathepsin axis as an upstream druggable node provides a mechanistic rationale for simultaneous multi-target cardioprotection and a route to mechanism-matched pharmacology in ischemic heart disease.

## 1. Introduction

Ischemia–reperfusion injury (IRI) represents a central paradox in coronary artery disease (CAD). Timely reperfusion salvages ischemic myocardium yet induces cardiomyocyte death that offsets therapeutic benefit [1]. This paradox reflects the coordinated activation of apoptosis, necroptosis, pyroptosis and ferroptosis [2,3,4]. During ischemia, ATP depletion and mitochondrial calcium overload prime cardiomyocytes for death [5,6]. Reperfusion amplifies these signals through ROS bursts and inflammatory cascades, executing diverse death programs [5,7].

Cathepsins are lysosomal proteases that integrate these death signals through lysosomal membrane permeabilization (LMP), inflammasome activation, and iron mobilization during ferroptosis [8,9]. In ischemic heart disease, they have been proposed to act as a shared proteolytic hub [9]. The cell death field has moved from an apoptosis-centric framework to a systems-level perspective. Apoptosis, necroptosis, pyroptosis, and ferroptosis no longer operate as isolated linear entities. They engage in molecular crosstalk and may converge into PANoptosis-like integrated-death platforms in some inflammatory and ischemic contexts [10,11,12,13]. We have recently shown that the necroptosis effector RIPK3 is actively secreted during myocardial ischemia–reperfusion and acts extracellularly as a damage-associated molecular pattern, coupling cell death to inflammatory amplification [14]. Furthermore, this extracellular RIPK3 predicts adverse outcomes after primary percutaneous coronary intervention (PCI) [14]. Myocardial ischemia–reperfusion injury (MIRI) is now viewed as an actively regulated process. Multiple death programs are coordinated in space and time.

Despite substantial advances, critical knowledge gaps remain. First, the temporal sequence of cathepsin-driven LMP across ischemic and reperfusion phases is poorly defined in cardiomyocytes and endothelial cells. Second, how this proteolytic event shifts cellular fate between distinct death programs in the heart remains unresolved, particularly in a cell-type-specific manner (cardiomyocytes versus endothelial cells). Third, compensatory activation between death modalities undermines single-target cardioprotective strategies, yet upstream convergence nodes have not been systematically exploited in cardiovascular medicine. We set out to connect these dots, using MIRI as the test case. In this review, we synthesize advances in molecular and translational cardiology to establish a unified framework linking lysosomal cathepsins with multimodal cell death in MIRI. By integrating cathepsin biology, cell fate regulation, endothelial dysfunction, and emerging therapeutic platforms, this review aims to provide a mechanistic basis and a translational path for mechanism-matched pharmacological cardioprotection in ischemic heart disease.

Throughout this review, evidence is classified into three tiers: (i) direct MIRI evidence, obtained from cardiomyocyte, cardiac-tissue, isolated-perfused-heart, or in vivo coronary occlusion/reperfusion models; (ii) cardiac ex vivo/in vitro evidence, obtained from cardiac-derived cells or tissues outside a complete MIRI paradigm; and (iii) extrapolated evidence, obtained from non-cardiac cells, tissues, or disease models. Extrapolated evidence is used only to fill mechanistic gaps and is explicitly identified as such.

Literature search strategy: PubMed/MEDLINE, Web of Science, and Embase were searched using combinations of the terms “cathepsin(s)”, “lysosomal membrane permeabilization”, “myocardial ischemia”, “myocardial infarction”, “ischemia-reperfusion injury”, “apoptosis”, “pyroptosis”, “ferroptosis”, “necroptosis”, “PANoptosis”, “endothelial injury”, “microvascular obstruction”, “no-reflow”, “biomarker”, “clinical trial”, and “therapeutic intervention”. English-language original research articles and reviews were eligible; seminal older studies were retained where they established foundational mechanisms. Evidence was prioritized in the following order: direct MIRI evidence, cardiac cellular or ex vivo evidence, and non-cardiac mechanistic evidence. The last category was used only to address unresolved mechanistic gaps and is explicitly identified as extrapolated evidence. Because this is a narrative review, the search strategy is reported in prose rather than as a PRISMA flow diagram.

## 2. Lysosomal Cathepsins and Lysosomal Membrane Permeabilization

### 2.1. Classification, Biosynthesis, and Proteolytic Activation

Cysteine cathepsins, such as cathepsin B, K, L, S, are synthesized as inactive zymogens, activated at lysosomal pH ~4.5–5.0, and regulated by cystatins. Cathepsin B (CTSB) is the most extensively implicated in death execution, owing to its dual exo/endopeptidase activity and partial cytosolic retention above pH 6.0 [8,9]. Cystatin C (CST3) deficiency accelerates atherosclerotic plaque progression, supporting the concept that cathepsin-cystatin imbalance compromises lysosomal integrity [15].

Although cathepsins were originally viewed mainly as executors of bulk protein degradation, accumulating evidence indicates they display considerable substrate specificity and participate in signaling events, receptor shedding, and regulated cell death pathways [15,16]. This functional diversity likely reflects restricted subcellular localization, pH-dependent conformational changes, and selective protein–protein interactions that together shape substrate choice and catalytic output [8,17].

### 2.2. Regulatory Mechanisms

Cathepsin activity depends on location and pH. Lysosomal targeting relies on mannose-6-phosphate receptor recognition. Disruption of this targeting causes missorting and secretion. Cysteine cathepsins exhibit maximal activity at pH 4.5–5.0. CTSB retains partial endopeptidase activity above pH 6.0, enabling cytosolic substrate cleavage after LMP [18,19]. CST3 forms reversible complexes with active cathepsins [16,20]. Under acidic conditions, CST3 dimerization abolishes its inhibitory function, acting as a pH-sensitive switch for localized activation [21]. Endogenous cystatins (cystatin C, CST3; stefins) set the cytosolic threshold for leaked cathepsin activity and constitute a first line of defense whose saturation determines whether limited LMP becomes lethal.

### 2.3. Lysosomal Membrane Permeabilization

LMP releases cathepsins from the acidic lumen into the neutral cytosol [22]. Oxidative stress and calcium overload destabilize the membrane [22]. In ischemia–reperfusion injury, excessive calpain activation further compromises lysosomal integrity [23,24]. The severity of LMP spans a spectrum: limited permeabilization permits partial enzyme leakage and may be followed by membrane repair, whereas massive rupture causes irreversible lysosomal content release and typically culminates in cell death. Once in the cytosol, cysteine cathepsins retain partial endopeptidase activity above pH 6.0 [18,19], enabling them to cleave specific substrates that propagate death signaling. Notably, cathepsins do not appear to activate procaspases directly; rather, they engage mitochondrial apoptosis [25], inflammasome platforms [26,27], and iron-mobilization pathways [28] through distinct proteolytic events. In fibroblasts and monocytes, LMP and cathepsin release function downstream of Bax/Bak activation and apoptosome assembly, serving as an amplification loop that accelerates executioner caspase activation without initiating apoptosis de novo [29]. It should be noted that LMP is rarely measured directly in vivo. Most evidence derives from galectin-3 puncta formation, cytosolic dextran release, or immunostaining of cytosolic cathepsins, each with distinct sensitivity limits [30].

### 2.4. Oxidative Stress, Calcium Overload, and the Regulation of Lysosomal Membrane Permeabilization During Reperfusion

Reperfusion ROS arise from interacting sources. These include an initial burst of superoxide from reverse electron transport at complex I, driven by oxidation of succinate accumulated during ischemia [31], as well as sustained production from uncoupled eNOS and NADPH oxidases [32,33]. Notably, NOX2 is prominent in endothelial cells and infiltrating leukocytes, whereas NOX4 is a major NADPH oxidase isoform in cardiomyocytes. This burst temporally coincides with the first wave of LMP, serving as a plausible trigger for lysosomal destabilization.

Two features make the lysosome a preferential target of this oxidative surge. As the cell’s iron-handling hub, it concentrates redox-active iron in an acidic lumen into which H_2_O_2_ freely diffuses, allowing Fenton-derived hydroxyl radicals to peroxidize the membrane from within [34,35]. In parallel, Ca^2+^ overload activates calpains that cleave lysosomal membrane–stabilizing proteins [23,24], creating a mutually reinforcing loop with ROS dysregulation. Once LMP occurs, the relationship becomes bidirectional: released cathepsins cleave Bid and degrade cytoprotective proteins, driving further mitochondrial ROS production and additional LMP. Ultimately, oxidative stress, lysosomal destabilization, and cathepsin release form an amplification loop rather than a linear pathway, although the complete operation of this loop in the reperfused cardiomyocyte remains to be directly demonstrated.

At the cardiomyocyte level, MIRI directly implicates apoptosis [36], NLRP3/GSDMD-mediated pyroptosis [2,37], ferroptosis [38,39], and RIPK3-dependent necroptosis [40,41]. However, direct cardiomyocyte evidence remains incomplete for several cathepsin-specific execution steps, including CTSB-mediated Bid cleavage [25,42], CTSB–NLRP3 binding [27,43], and CTSB-mediated ferritin degradation [44,45]. In isolated perfused hearts, reperfusion-associated cathepsin release has been directly linked to contractile dysfunction and infarct size [46]. In rodent and large-animal in vivo models, death-pathway activation and therapeutic modulation can be evaluated in an integrated cardiac and systemic context [37], although species-specific translation remains limited. At the endothelial level, direct MIRI-specific evidence is less complete than at the cardiomyocyte level, and several detailed mechanisms have been characterized in non-coronary endothelial models [47,48]. These distinctions are developed in Section 3, Section 4 and Section 5.

## 3. Cathepsins as Orchestrators of Regulated Cell Death

### 3.1. Cathepsins and Apoptosis

Apoptosis represents the most extensively mapped death program linked to lysosomal cathepsin release. Direct MIRI evidence supports a role for CTSB in cardiomyocyte apoptosis and infarct limitation; however, direct demonstration of CTSB-mediated Bid cleavage in reperfused cardiomyocytes remains incomplete [25,36,42]. Once translocated to the cytosol, CTSB cleaves the pro-apoptotic Bcl-2 family protein Bid to generate truncated Bid (tBid). tBid translocates to mitochondria and promotes outer membrane permeabilization [42]. CTSL and CTSS may similarly process Bid and antagonize anti-apoptotic Bcl-2 homologues such as Bcl-xL and Mcl-1 [42]. Following mitochondrial outer membrane permeabilization, cytochrome c enters the cytosol, binds Apaf-1, and activates caspase-9, which cleaves executioner caspases-3 and -7 [49]. CTSD can also contribute to this axis by cleaving Bid at distinct sites or promoting Bax activation under specific conditions [50]. In most contexts, this proteolytic route amplifies apoptosis that has already been primed by upstream signals. It is quantitative rather than qualitative [29].

### 3.2. Cathepsins and Pyroptosis

NLRP3 inflammasome activation and GSDMD-mediated pyroptosis are directly supported in MIRI models, including large-animal studies [2,37]. By contrast, direct CTSB–NLRP3 binding has been demonstrated in macrophage and cholesterol-crystal systems and remains to be shown in cardiomyocytes [27,43]. CTSB is the family member most consistently implicated in NLRP3 inflammasome activation across diverse stimuli [27]. CTSB binds directly to NLRP3 after LMP. This transient complex licenses inflammasome assembly and caspase-1 recruitment [27]. Once activated, caspase-1 cleaves gasdermin D (GSDMD) to trigger pyroptosis, and processes pro-IL-1β and pro-IL-18 into mature forms [51,52]. Cholesterol crystals induce the same axis in atherosclerotic macrophages [27,43]. Cardiac cells likely use similar machinery, though direct demonstration of CTSB-NLRP3 binding in cardiomyocytes remains to be shown.

### 3.3. Cathepsins and Ferroptosis

Ferroptosis is directly implicated in MIRI [38,39], but CTSB-mediated ferritin degradation and iron mobilization are supported mainly by non-cardiac studies [44,45]. This mechanism is therefore classified as extrapolated evidence. CTSB has recently been implicated in ferroptotic cell death. Both pharmacological inhibition and genetic deletion of this protease attenuate ferroptosis induced by erastin and RSL3 [44,45]. CA-074Me can lose CTSB selectivity in intact cells because it additionally inhibits cathepsin L within living cells [53]. Consequently, CA-074Me-only experiments are interpreted here as demonstrating a CTSB-predominant effect rather than a necessarily CTSB-exclusive one. Notably, these studies combined CA-074Me with genetic loss-of-function approaches, and the inhibitor and genetic phenotypes were concordant, which mitigates, though does not eliminate, the selectivity concern raised in Section 7.1 for intact-cell pharmacology. Under ferroptotic stress, CTSB promotes ferritin degradation and iron mobilization, probably through ferritinophagy. Iron-loaded ferritin is delivered to lysosomes, where cathepsins degrade the ferritin shell and release stored iron. The liberated Fe^2+^ drives lipid peroxidation through Fenton chemistry [44,45,54]. In the myocardium, this process may be exacerbated by concurrent downregulation of glutathione peroxidase 4 (GPX4), which leaves cardiomyocytes unable to clear accumulating lipid reactive oxygen species [38]. Whether lysosomal iron release alone is sufficient to trigger lethal ferroptosis in all cardiomyocyte contexts remains uncertain, though arachidonate 15-lipoxygenase (Alox15) derived 15-Hydroperoxyeicosatetraenoic acid (15-HpETE) has been shown to promote ferroptosis during myocardial ischemia–reperfusion [39]. Even with these caveats, direct demonstration that cardiomyocyte ferritin is a CTSB substrate under ischemia–reperfusion conditions remains outstanding.

### 3.4. Cathepsins and Necroptosis

Necroptosis is driven by RIPK1, RIPK3, and the pseudokinase MLKL. Necroptosis itself is directly implicated in MIRI [40,55], but direct molecular links between cathepsins and RIPK1/RIPK3/MLKL signaling have not been demonstrated in cardiac cells. The proposed cathepsin contribution is therefore indirect. Lysosomal disruption may amplify necroptotic signaling through cathepsin release. In ischemic brain injury, combined inhibition of CTSB and caspase-3 provides greater protection than either alone [56], suggesting that lysosomal proteases can interact with necroptotic machinery in ischemic tissue. Direct molecular links between cathepsins and the necrosome remain poorly defined in cardiac cells. Two indirect routes are plausible. Lysosomal rupture releases iron and ROS that could, hypothetically, oxidize RIPK3 cysteine residues and thereby modulate kinase activity, although this mechanism has not been demonstrated to our knowledge. Alternatively, CTSB-mediated Bid cleavage generates mitochondrial ROS that secondarily sensitizes cells to necroptotic execution. Beyond its intracellular execution role, RIPK3 is actively secreted from cardiomyocytes during ischemia–reperfusion and acts extracellularly as a damage-associated molecular pattern via the RAGE-CaMKII pathway. This signaling axis amplifies inflammatory cascades and cell death across cardiac, vascular, and immune cells. Plasma RIPK3 measured 6 h after PCI independently predicted long-term major adverse cardiovascular events in patients with acute myocardial infarction [14]. Direct physical coupling between cathepsins and the RIPK3-MLKL axis has not been demonstrated in cardiomyocytes. Accordingly, cathepsins are best viewed as potential modulators rather than established executioners of necroptosis in MIRI.

### 3.5. Cathepsins and Autophagy

Autophagic flux requires lysosomal degradation for quality control [56]. Cathepsin L (CTSL) deficiency impairs TFEB activation and blocks autophagic flux, triggering the accumulation of p62 and ubiquitinated proteins. This metabolic collapse, characterized by reduced ATP production and elevated oxidative stress, ultimately leads to apoptotic cell death [57,58,59]. When lysosomal proteolysis is disrupted more generally, cargo degradation stalls and the resulting proteotoxic stress shifts the balance from survival to cell death during ischemia–reperfusion [60]. In experimental MIRI, impaired autophagosome clearance directly contributes to cardiomyocyte death [61]. This contrasts with the death-promoting role of cytosolic cathepsins. Specifically, impaired lysosomal degradation sensitizes cells to LMP, whereas released cathepsins actively execute death. These represent two mechanistically distinct consequences of lysosomal dysfunction. Impaired autophagic flux and lysosomal proteolysis are supported by cardiac I/R and cardiac-cell studies [57,58,61], but these findings should be distinguished from cytosolic cathepsin-mediated death execution.

### 3.6. Inter-Modal Crosstalk and Convergence

Cathepsins released after LMP can engage several death pathways simultaneously. The same pool of cathepsins may cleave Bid to amplify apoptosis, activate NLRP3 to trigger pyroptosis, or degrade ferritin to fuel ferroptosis [27,42,44]. The dominant death modality is determined by the specific cathepsin isoforms released and the severity of LMP. This decision is further modulated by the cellular metabolic context, particularly the availability of iron stores, the expression level of Bcl-2 family proteins, and the priming state of the NLRP3 inflammasome.

**Figure 1:**
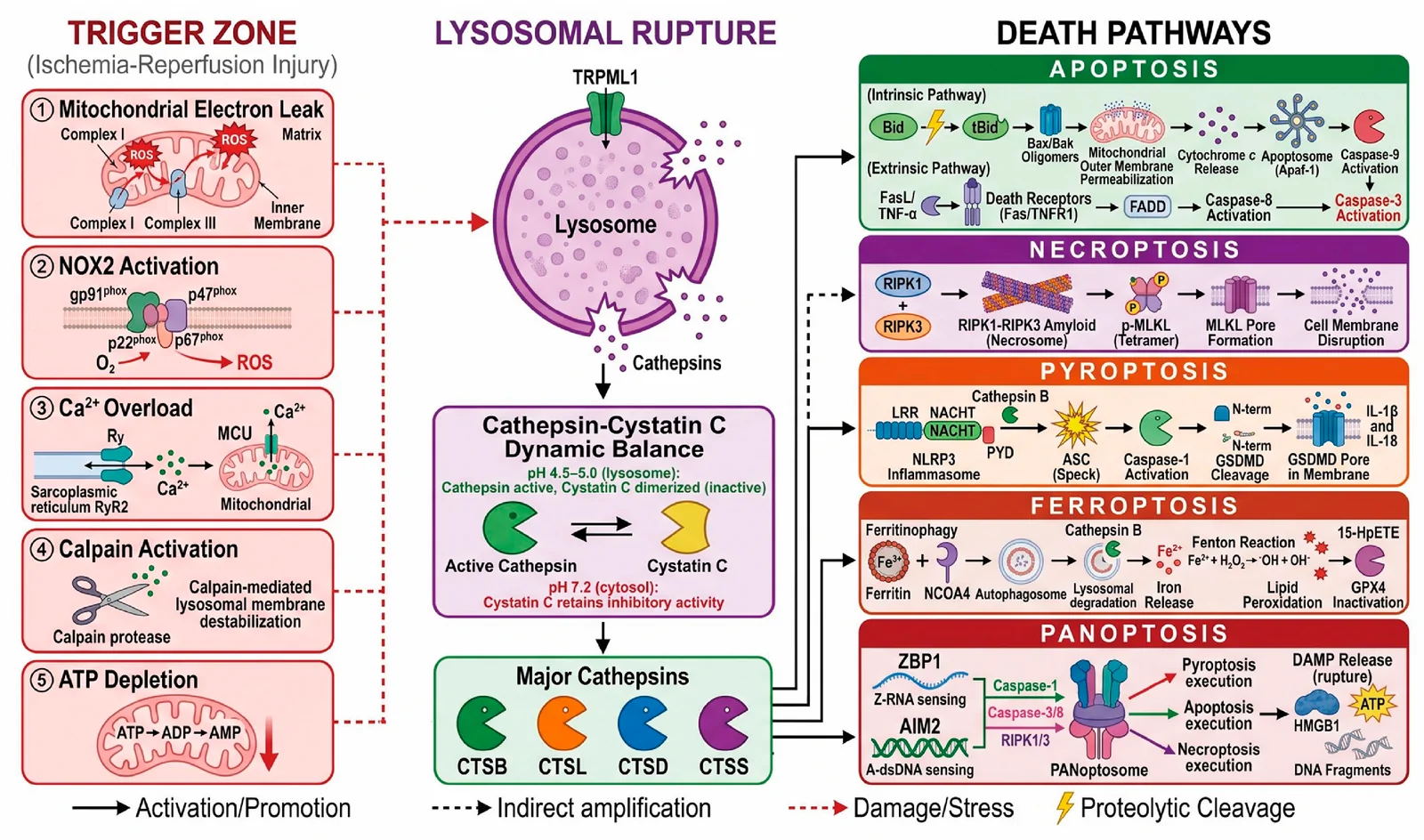
The lysosomal-cathepsin axis as a proteolytic nexus for multi-modal regulated cell death in myocardial ischemia–reperfusion injury. The corresponding temporal sequence—ischemic priming, early-predominant apoptosis and pyroptosis, intermediate-to-late necroptosis and ferroptosis, and later integrated death—is described in Section 4 and is model-dependent. Ischemia–reperfusion triggers (left) induce lysosomal membrane permeabilization (LMP), releasing cathepsins cathepsin B (CTSB), cathepsin L (CTSL), cathepsin D (CTSD) and cathepsin S (CTSS) into the cytosol where cystatin C (CST3) modulates proteolytic activity (center). Released cathepsins simultaneously engage five death programs (right): apoptosis, necroptosis, pyroptosis, ferroptosis, and PANoptosis.

PANoptosis integrates pyroptosis, apoptosis, and necroptosis through ZBP1- or RIPK1-PANoptosome platforms. CTSB contributes to this convergence by activating NLRP3 and cleaving Bid, lowering the threshold for combined death execution [27,42]. The severity of LMP and metabolic context determine whether CTSB triggers isolated modalities or converges on PANoptosis (Figure 1). ZBP1-dependent integrated cell death has direct MIRI evidence [62,63], whereas assembly of a canonical PANoptosome has not been directly demonstrated in cardiomyocytes. Ferroptosis remains outside the canonical PANoptosome. Instead, its integration here reflects shared upstream triggers (LMP, iron mobilization) rather than a common execution platform.

Taken together, apoptosis, pyroptosis, ferroptosis, and necroptosis are directly implicated in MIRI, although the depth and specificity of evidence differ among these modalities. In contrast, direct demonstration of cathepsin-mediated execution events in cardiomyocytes under I/R conditions remains incomplete. CTSB-dependent apoptosis is supported by MIRI pharmacological studies, but direct CTSB-mediated Bid cleavage in reperfused cardiomyocytes remains to be demonstrated. CTSB–NLRP3 binding has been shown in macrophage and cholesterol-crystal systems but not in cardiomyocytes. CTSB-mediated ferritin degradation and iron mobilization during ferroptosis are supported mainly by non-cardiac studies, whereas the cathepsin–necroptosis connection remains indirect. ZBP1-dependent integrated cell death has direct MIRI evidence, but assembly of a canonical PANoptosome has not been demonstrated in cardiomyocytes. Defining these cathepsin-specific interactions in cardiomyocytes under I/R conditions is therefore an important unresolved question.

## 4. The Cathepsin Nexus in Myocardial Ischemia–Reperfusion Injury

### 4.1. Pathophysiology and Temporal Phases

Coronary occlusion halts oxidative phosphorylation, leading to severe ATP depletion. The resulting cytosolic Na^+^ and Ca^2+^ overload disrupts mitochondrial and lysosomal homeostasis and primes cardiomyocytes for death [1]. ALOX15 generates 15-HpETE during ischemia, sensitizing cardiac mitochondria to ferroptotic stress upon reperfusion. TFEB-mediated lysosomal biogenesis initially supports metabolic adaptation, but impaired lysosomal proteolysis or blocked autophagic flux can become detrimental during sustained stress [58,64,65,66,67]. Reperfusion rapidly reintroduces oxygen and triggers mitochondrial ROS production, NADPH oxidase activation, calcium overload, and calpain-mediated injury [24,32,33]. CTSL release into the cytosol peaks at approximately 20 min after reflow, correlating with contractile dysfunction [46]. Serum CST3 rises acutely in STEMI patients, reflecting protease–inhibitor imbalance, although renal function must be considered when interpreting circulating levels [68]. Neutrophils infiltrate the myocardium within hours, promoting microvascular obstruction and extending ischemic injury [69,70,71].

### 4.2. Cell Death Modalities in Myocardial Ischemia–Reperfusion Injury

Ferroptosis in MIRI: Ferroptosis contributes particularly to intermediate and late reperfusion injury and is closely linked to metabolic state and iron availability. It is executed through iron-dependent lipid peroxidation rather than by caspases or pore-forming proteins [72]. The ALOX15/15-HpETE axis promotes this late-phase ferroptosis through PGC-1α ubiquitination and consequent impairment of mitochondrial biogenesis [39].

At the metabolic level, ferroptosis in MIRI reflects the interplay between iron trafficking and lipid peroxidation. NCOA4-mediated ferritinophagy delivers ferritin to lysosomes, where CTSB may degrade the ferritin shell and mobilize free iron [44,73]. However, direct demonstration that cardiomyocyte ferritin is a CTSB substrate under ischemia–reperfusion conditions remains lacking; this component of the mechanism is therefore regarded as extrapolated evidence. This labile iron drives peroxidation of PUFA-containing phospholipids through lipoxygenase activity and non-enzymatic Fenton reactions [4,74]. GPX4, the principal enzyme that reduces phospholipid hydroperoxides to harmless alcohols, normally acts as a critical brake on ferroptotic execution; its catalytic activity depends on selenocysteine at the active site [75,76]. GPX4 protein levels decline during ischemic injury in murine myocardial infarction models, and pharmacological inhibition of ferroptosis with ferrostatin-1 reduces infarct size [38]. The later appearance of ferroptosis relative to acute necrotic or apoptotic death suggests a potential therapeutic window, although its timing is strongly model-dependent [38,39].

Pyroptosis in MIRI: Consistent with the upstream role of CTSB in NLRP3 activation discussed above, pharmacological blockade of the CTSB-NLRP3 axis has been tested in myocardial infarction models. CTSB inhibition after myocardial infarction attenuates NLRP3 inflammasome activation and limits adverse remodeling [77]. The selective NLRP3 inhibitor MCC950 reduces infarct size and preserves cardiac function when administered before reperfusion in a porcine model [37] and reduces myocardial fibrosis and improves post-infarction remodeling in permanently occluded mouse hearts [78]. Pyroptosis is particularly prominent during early reperfusion, when inflammatory signaling is most intense [37,77]. However, direct CTSB–NLRP3 binding has not yet been demonstrated in cardiomyocytes; the binding mechanism is currently supported by macrophage and cholesterol-crystal studies [27,43]. The noncanonical pathway contributes in settings of intense oxidative stress, where caspase-11 cleaves GSDMD and promotes IL-18 release, thereby broadening the pyroptotic burden beyond the canonical NLRP3 route [2,79]. Genetic deletion of GSDMD specifically in cardiac cells significantly reduces infarct size and improves survival in experimental MIRI [2].

Apoptosis and Necroptosis in MIRI: Apoptosis is triggered through intrinsic and extrinsic pathways during ischemia–reperfusion. CTSB release can directly engage this pathway through Bid cleavage, but direct demonstration of CTSB-mediated Bid cleavage in reperfused cardiomyocytes remains incomplete [25,36,42]. Pharmacological inhibition of CTSB with CA-074Me attenuates infarct expansion in experimental MIRI [36]. Because CA-074Me can lose CTSB selectivity in intact cells, this finding is interpreted as CTSB-predominant rather than necessarily CTSB-exclusive. Necroptosis is directly implicated in MIRI through RIPK1/RIPK3/MLKL signaling [40,55]. RIPK3 phosphorylates CaMKIIδ, promoting mitochondrial permeability-transition-pore opening and cardiomyocyte death [41]. In contrast, direct molecular links between cathepsins and the necrosome have not been established in cardiac cells; cathepsins are therefore best regarded as potential modulators rather than established executioners of necroptosis in MIRI.

### 4.3. PANoptosis and Integrated Cell Death Networks

Death modalities in MIRI are not isolated phenomena but components of an integrated cell death network. Necroptosis and PANoptosis-associated features emerge predominantly during later reperfusion and post-reperfusion remodeling. Their contribution is shaped by DAMP release from dying cells, which primes RIPK3-MLKL signaling and PANoptosome assembly in neighboring viable cardiomyocytes [41,80,81]. As detailed above, ferroptosis operates in parallel rather than as a constituent of PANoptosis, yet it shares LMP and cathepsin release as a common upstream contributor [82,83]. In infarcted myocardium, PANoptosis execution is synchronized with intense inflammatory cell infiltration and cytokine storms, creating a feed-forward loop that extends injury beyond the initial ischemic territory [82,84]. CTSB may contribute to integrated death execution through NLRP3 activation and Bid cleavage, two established substrates that interface with PANoptosome assembly. Cardiomyocyte-specific ZBP1 deficiency ameliorates PANoptosis-like integrated death and reduces infarct size, and Prussian blue nanozymes targeting PANoptosome components provide direct MIRI evidence for therapeutic modulation of this network [62,63]. Nevertheless, assembly of a canonical PANoptosome has not been directly demonstrated in cardiomyocytes. PANoptosis is therefore used here as a conceptual framework for integrated death rather than as a fully established cardiomyocyte execution platform.

### 4.4. Spatiotemporal Coordination of Death Modalities

MIRI proceeds through overlapping temporal phases rather than through a single uniform death pathway. Early reperfusion is characterized by ATP depletion, calcium overload, mitochondrial injury, and rapid lysosomal cathepsin release. This early phase favors apoptosis and NLRP3/GSDMD-mediated pyroptosis, although apoptosis can also persist into later phases [2,36]. With prolonged reperfusion, ALOX15-derived lipid peroxides, declining GPX4 antioxidant capacity, inflammatory amplification, endothelial injury, and RIPK3-dependent signaling shift the injury profile toward ferroptosis, necroptosis, and PANoptosis-like integrated death [39,41,46,62] (Table 1). Figure 1 depicts the spatial organization of the lysosomal-cathepsin nexus rather than its temporal sequence. Ischemia primes cardiomyocyte death through ATP depletion, Ca^2+^ overload, mitochondrial dysfunction, and ALOX15-dependent lipid signals. Within minutes to approximately 2 h after reperfusion, ROS and Ca^2+^ overload promote LMP and cathepsin release, with apoptosis and NLRP3/GSDMD-mediated pyroptosis predominating. Between approximately 2 and 6 h, inflammatory amplification, endothelial injury, necroptosis, and microvascular obstruction become increasingly prominent, while ferroptosis contributes according to iron availability and GPX4 capacity. During later reperfusion and remodeling, RIPK3/MLKL signaling, PANoptosis-like integrated death, and persistent ferroptotic injury may extend tissue damage. These temporal boundaries overlap substantially and vary with species, ischemia duration, reperfusion interval, and experimental model.

### 4.5. Experimental Models: Strengths, Limitations, and Interpretive Caveats

The apparent contribution of any cathepsin to MIRI is conditioned by the experimental system in which it is measured (Table 2). Ischemia duration shapes ATP preservation, ionic collapse, mitochondrial injury, and the relative prominence of apoptosis, pyroptosis, necroptosis, and ferroptosis. Brief ischemia with residual ATP can favor apoptotic execution, whereas prolonged ischemia with severe ATP depletion shifts the balance toward necrotic and inflammatory modalities, including ferroptosis and necroptosis [47]. Reperfusion duration determines whether a study captures the early ROS-driven death wave or the later inflammatory and remodeling phase [1,32,33]. Consequently, effect sizes and pathway rankings are not directly comparable across studies unless the ischemia and reperfusion protocols are considered together.

Cellular and species context are equally important. Isolated cardiomyocyte hypoxia/reoxygenation systems permit mechanistic and genetic experiments but lack blood flow, endothelial interactions, platelets, complement, and circulating leukocytes. Langendorff-perfused hearts preserve tissue architecture and contractile function but usually exclude blood-borne inflammatory cells. In vivo rodent models retain systemic inflammatory responses but differ from human hearts in heart rate, coronary anatomy, collateral circulation, and infarct geometry. Porcine models more closely recapitulate human coronary anatomy and infarct geometry and are valuable for therapeutic translation, whereas human samples provide direct clinical relevance but cannot by themselves establish causality. These caveats explain why cathepsin-inhibitor efficacy may differ between isolated-heart and in vivo studies and why large-animal and human studies remain a critical translational evidence gap.

## 5. Endothelial and Microvascular Dysfunction

### 5.1. Cathepsin-Mediated Endothelial Cell Death

The vascular endothelium is both a target and a mediator of IRI [85]. During reperfusion, endothelial cells experience intense oxidative and inflammatory stress. Oxidants arise from the endothelium itself through uncoupled eNOS and NOX2, as well as from adherent leukocytes. As detailed in Section 2.4, these oxidants converge on the lysosomal compartment, and the iron-rich membrane is peroxidized from within by Fenton chemistry [32,33,34,35]. Lysosomal membranes undergo permeabilization in these cells. Consequently, CTSB leaks into the cytosol and promotes NLRP3 inflammasome assembly, caspase-1 activation, and GSDMD cleavage [27,47]. This cascade has been demonstrated in a Kawasaki disease vasculitis model [47]. However, this constitutes extrapolated evidence because direct demonstration in the reperfused coronary microvasculature is still pending.

Pharmacological inhibition of CTSB with CA-074Me attenuates this endothelial death and reduces vascular inflammatory infiltration. This effect is interpreted as CTSB-predominant rather than CTSB-exclusive given the reduced selectivity of the compound in intact cells [53]. Through this axis, endothelial LMP may couple the reperfusion ROS burst to inflammasome activation, junctional disruption, and barrier failure. These processes represent the upstream events of microvascular obstruction and no-reflow, as discussed in Section 5.3. Direct measurements of endothelial LMP during myocardial reperfusion in vivo remain limited, and this endothelial arm of the lysosomal pathway warrants dedicated study.

### 5.2. Endothelial–Immune Cell Interactions

Communication between endothelial cells and infiltrating immune cells amplifies ischemia–reperfusion injury. Following reperfusion, the injured coronary endothelium exhibits a rapid upregulation of P-selectin and intercellular adhesion molecule-1 (ICAM-1). These adhesion molecules orchestrate the sequential steps of neutrophil rolling, firm adhesion, and eventual transmigration into the ischemic myocardium [86]. Combined blockade of P-selectin and ICAM-1 preserves myocardial blood flow and attenuates neutrophil accumulation [87]. Cathepsin S (CTSS) degrades elastin and extracellular matrix components, facilitating immune cell transmigration [88]. Beyond matrix degradation, CTSS impairs cholesterol efflux from macrophage foam cells by degrading apolipoprotein A-I. This promotes lipid retention and in turn sustains plaque inflammation [89,90].

Upon reperfusion, CTSB released from endothelial cells with compromised lysosomal integrity activates the NLRP3 inflammasome, leading to GSDMD cleavage and pore formation. This pyroptotic death disrupts endothelial integrity, increases vascular permeability, and promotes leukocyte extravasation. The resulting edema and inflammation compress capillaries and impair microvascular perfusion [91,92,93]. Endothelial pyroptosis thereby links cathepsin activity directly to microvascular obstruction, a critical determinant of infarct expansion and adverse remodeling. In STEMI patients, circulating NET markers peak after primary PCI and are associated with microvascular obstruction and larger infarct size [94]. Endothelial protection is also emerging as the mechanistic basis of non-pharmacological benefit in coronary heart disease. For example, cardiac rehabilitation pathways preserve endothelial function through intersecting molecular routes [95].

### 5.3. Microcirculatory Impairment and the No-Reflow Phenomenon

The no-reflow phenomenon occurs in 5–50% of STEMI patients after primary PCI, depending on the definition applied. Angiographic definitions such as TIMI flow grade or myocardial blush grade report 11–29% in cohort studies, whereas cardiac magnetic resonance-defined microvascular obstruction detects higher rates because it captures anatomically compromised microvasculature that angiography cannot resolve [96]. The mechanisms are multifactorial and include endothelial cell swelling with luminal obliteration, microvascular embolization by thrombus and atheromatous debris, interstitial edema with capillary compression, and neutrophil-platelet aggregation causing microvascular plugging.

Cathepsins contribute to no-reflow through multiple mechanisms. CTSS degrades elastin and basement membrane components, facilitating immune cell transmigration and promoting microvascular injury [88]. CTSB-driven NLRP3 inflammasome activation in endothelial cells leads to GSDMD-mediated pyroptosis and IL-1β release, promoting microvascular dysfunction [48,79,97]. In free-fatty-acid-challenged endothelium, lysosomal compromise releases CTSB, which activates the NLRP3 inflammasome and increases monolayer permeability through GSDMD-dependent pore formation, and pharmacological blockade of inflammasome assembly attenuates this barrier disruption [48,98].

Neutrophil extracellular traps released during reperfusion further obstruct coronary microvessels. NET burden in culprit-artery thrombi correlates positively with infarct size and negatively with ST-segment resolution. Notably, the inverse correlation between local DNase activity and NET burden suggests that enzymatic NET degradation may limit myocardial injury [99]. The structural components of no-reflow exhibit differential reversibility. While endothelial swelling and edema may be reversible with vasodilator therapy, microthrombi are structurally stable and largely resistant to pharmacological resolution. This dichotomy underscores the critical need for preventive strategies over reactive treatment.

## 6. Biomarkers of Cathepsin Activity and Multi-Modal Cell Death

### 6.1. Cathepsins as Circulating and Imaging Biomarkers

Serum Cathepsin Levels: CTSS serves as a biomarker of atherosclerotic burden. Elevated CTSS levels are associated with increased cardiovascular mortality in population cohorts [100]. Serum CTSL is significantly elevated in acute coronary syndrome patients compared with stable angina patients [46,101]. In acute heart failure, CST3 rises acutely and predicts mortality independent of renal function [102,103]. Interpretation of circulating cathepsin concentrations requires attention to the choice between activity-based probes and immunoassays. Renal function is another critical factor. It confounds cystatin C and, indirectly, cathepsin levels. Meanwhile, death-pathway effectors themselves are entering the biomarker arena. Plasma RIPK3 measured 6 h after primary PCI independently predicted long-term MACE [14] and circulating GSDMD associated with microvascular obstruction and intramyocardial hemorrhage after STEMI [104].

Molecular Imaging of Cathepsin Activity: Cathepsin activity can be visualized in vivo using molecular imaging probes. Fluorescently labeled substrates detect CTSB activity in atherosclerotic plaques with high specificity [105]. Photoacoustic imaging using lipid-based probes achieves tissue penetration exceeding 1.0 cm, detecting CTSB in plaques [106]. These findings support potential translational use in catheter-based plaque assessment.

### 6.2. Biomarkers of Multi-Modal Cell Death

Ferroptosis Biomarkers: Iron metabolism and lipid peroxidation products serve as potential ferroptosis biomarkers. Ferritin levels may reflect iron stores. The ALOX15-derived metabolite 15-HpETE may serve as a specific early indicator in ischemic myocardium [38]. GPX4 protein levels decline during ischemic injury in murine models. This downregulation correlates with ferroptotic death extent, supporting GPX4 as a functional biomarker [38]. Serum ferritin and non-transferrin-bound iron reflect the labile iron pool available to catalyze lipid peroxidation.

Pyroptosis Biomarkers: Pyroptosis biomarkers reflect inflammasome activation and GSDMD execution. In STEMI patients, serum GSDMD and IL-18 levels were significantly elevated after percutaneous coronary intervention, suggesting active pyroptotic execution during reperfusion [107]. IL-1β and IL-18 mature through caspase-1 cleavage within hours of acute coronary syndrome onset. Circulating ASC specks, oligomerized ASC released from activated inflammasomes, represent a structural biomarker of inflammasome activity that may complement cytokine-based pyroptosis assessment [108].

Necroptosis Biomarkers: Necroptosis biomarkers reflect RIPK3 kinase activation and MLKL phosphorylation. p-RIPK3 and p-MLKL are elevated in ischemic myocardium in experimental models. They correlate with necroptosis severity, suggesting utility as dynamic indicators of necroptotic burden [109,110,111].

PANoptosis-Associated Signatures: A PANoptosis biomarker panel would integrate pyroptosis, apoptosis, and necroptosis markers. Combined measurement of GSDMD, cleaved caspase-3, and p-MLKL could provide a composite readout of integrated cell death activity. Formal validation in MIRI cohorts remains to be reported. These candidate markers differ in validation status across three tiers. First, clinically validated and routinely available assays include cystatin C. Second, analytes measurable on validated immunoassay platforms have circulating CTSL, CTSS, GSDMD, IL-1β, and IL-18, although their MIRI-specific evidence remains observational or preclinical. Third, research-grade markers are restricted to specialized settings and include ASC specks, 15-HpETE with related lipid-peroxidation panels, and phospho-RIPK3/phospho-MLKL tissue signatures. Table 3 presents these tiers explicitly in the “Clinical Platform Readiness” column (Table 3).

### 6.3. Multi-Omics Integration and Clinical Translation

Multi-Omics Signatures: Single biomarker approaches capture limited facets of complex ischemia–reperfusion pathophysiology. Multi-omics integration offers more comprehensive characterization by simultaneously assessing protein activity and metabolic states. In MIRI, ALOX15-derived 15-HpETE and related eicosanoids have been identified as specific ferroptosis-associated metabolites [39]. More comprehensive metabolomic profiling in STEMI patients undergoing primary PCI has identified a panel of three plasma metabolites, including pentadecanoic acid, linoleoyl carnitine, and 1-linoleoylglycerophosphocholine. When validated against peak troponin and creatine kinase, this panel can discriminate small from large infarct sizes with a predictive accuracy exceeding 80% [114]. Machine learning algorithms have been applied to integrate multi-omics data for acute myocardial infarction risk prediction. Ensemble approaches combining transcriptomic and clinical data achieved area-under-the-curve values of 0.874 in training cohorts and 0.756–0.780 in independent validation sets, outperforming single-feature models [115].

Clinical Validation: The translation of multi-omics biomarkers into clinical practice requires analytical standardization and prospective validation. Assays for emerging biomarkers such as GSDMD N-terminal fragment, p-MLKL, and 15-HpETE currently lack clinical-grade precision and inter-laboratory standardization. Serum GSDMD levels, measurable by ELISA platforms, have shown independent prognostic value in STEMI patients [116]. Such biomarkers could enable risk stratification and therapeutic monitoring during the acute phase.

Biomarker-guided risk stratification must demonstrate improved outcomes in prospective trials. The CANTOS trial established that targeting IL-1β signaling in patients with elevated hs-CRP reduces recurrent cardiovascular events [113]. Low-dose colchicine showed cardiovascular protection in post-myocardial infarction and chronic coronary syndrome populations [117,118]. These trials offer precedent that modulating downstream inflammatory mediators released during pyroptosis can improve cardiac prognosis, yet they did not stratify patients by GSDMD, p-MLKL, or 15-HpETE levels. Prospective studies randomizing treatment according to multi-modal cell death biomarker panels are necessary to validate precision-medicine approaches.

## 7. Therapeutic Strategies and Translational Challenges

### 7.1. Cathepsin-Targeted Interventions

Broad-spectrum cysteine cathepsin inhibitors, including E64 and its derivatives, non-selectively block cysteine protease activity and hereby preventing cytosolic cathepsin-mediated Bid cleavage [25]. However, these compounds lack isoform selectivity and additionally inhibit unrelated cysteine proteases such as calpains, disrupting physiological proteolysis in antigen presentation and muscle homeostasis. These off-target effects produce dose-limiting toxicities that have precluded clinical development.

The CTSB-selective inhibitor CA-074Me attenuates infarct size and reduces serum cardiac troponin I in mouse MIRI models, limiting caspase-3 activation [36]. This constitutes direct MIRI evidence at the pharmacological level. However, the underlying CTSB–Bid cleavage step is established most directly in non-cardiac systems and is only inferred in cardiomyocytes (Section 3.1). In STEMI patients, serum CTSL rises immediately upon reperfusion and correlates with infarct size and contractile dysfunction. The selective CTSL inhibitor CAA0225 reduces infarct size in preclinical models [46]. The circulating CTSL association is a direct clinical observation but remains correlative, whereas CAA0225 efficacy is preclinical. No cathepsin inhibitor has yet been tested in reperfused patients. Odanacatib, a CTSK inhibitor, was discontinued due to an elevated risk of cardiovascular events observed during osteoporosis trials [119]. By contrast, selective CTSS inhibition shows a more favorable profile in chronic atherosclerosis models [120]. Selective inhibitors must therefore be matched to the specific cathepsin implicated in each death pathway. The pharmacological properties of cathepsin inhibitors deserve emphasis. Epoxysuccinyl compounds such as E64d (aloxistatin) reached clinical trials for muscular dystrophy but failed for efficacy rather than safety, indicating that broad cysteine cathepsin blockade is tolerable in humans. CA-074 is highly selective for CTSB in vitro, yet its membrane-permeant methyl ester CA-074Me partially loses this selectivity in intact cells—a caveat for interpreting preclinical MIRI studies. Most cathepsin inhibitors are peptidomimetics with modest oral bioavailability. However, their lysosomotropic accumulation in acidic compartments can be exploited for target engagement at the site of action.

### 7.2. Modulators of Individual Death Pathways

Ferroptosis inhibition has shown cardioprotective potential in preclinical models. First-generation radical-trapping antioxidants suffer from poor metabolic stability and aqueous solubility. UAMC-3203 attenuates post-resuscitation myocardial dysfunction, reduces lipid peroxidation, and preserves GPX4 expression in rat cardiac arrest models [121]. Iron chelation is an alternative strategy, though clinical translation has proven challenging. Iron chelation nevertheless has a clinical precedent in the heart. For instance, dexrazoxane is approved to prevent anthracycline cardiotoxicity, providing a regulatory and dosing framework that ferroptosis-directed chelation strategies can build upon [122]. These agents act downstream of the cathepsin–ferritinophagy axis, whose execution steps are demonstrated predominantly in non-cardiac cells (Section 3.3). Therefore, their cardioprotective efficacy does not by itself validate the cathepsin–ferroptosis coupling.

The CANTOS trial validated IL-1β suppression in atherosclerotic disease [113,123]. Colchicine attenuates NLRP3 inflammasome assembly through microtubule inhibition and has achieved clinical validation in chronic coronary disease [117,118,124]. In myocardial infarction models, the selective NLRP3 inhibitor MCC950 (CRID3) reduces infarct size and preserves cardiac function when administered before reperfusion [37]. NLRP3 is a directly validated MIRI target; however, the upstream CTSB–NLRP3 coupling derives from macrophage and atherosclerosis models and remains to be demonstrated in cardiomyocytes (Section 3.2). The oral NLRP3 inhibitor dapansutrile (OLT1177) has shown safety and preliminary efficacy in a phase 1B trial in patients with systolic heart failure and is currently the most clinically advanced selective NLRP3 inhibitor relevant to cardiovascular indications [125]. Despite this progress, the timing of NLRP3 inhibition appears critical. Recent analyses highlight that early intervention during the acute inflammatory burst may be necessary, whereas delayed administration may miss the therapeutic window [126]. More selective NLRP3 inhibitors suitable for acute peri-procedural administration remain in development, and their evaluation in STEMI cohorts is a priority for future trial design [127] (Table 4).

### 7.3. Advanced Delivery Systems and Combination Therapies

Nanomedicine offers solutions to specificity and delivery challenges. Targeted nanoparticles can deliver cathepsin inhibitors to ischemic myocardium. Preclinical studies have demonstrated that polymeric nanoparticles loaded with the antioxidant ginsenoside Rg3 achieve ROS-triggered release at the injury site, thereby reducing infarct size and improving cardiac function [134]. PLGA nanoparticles demonstrate preferential accumulation in ischemic myocardium [135].

Multi-modal cell death demands combination approaches. Suppressing one modality triggers compensatory activation of alternatives. Cytosolic CTSB degrades ferritin to mobilize free iron that drives lipid peroxidation. Simultaneous inhibition of CTSB and ferroptosis addresses both upstream trigger and downstream executioner [44,45]. Disruption of the NCOA4–FTH1 interaction blocks ferritinophagy-derived iron mobilization as an alternative strategy [136].

Targeting PANoptosis requires simultaneous modulation of pyroptosis, apoptosis, and necroptosis. In MIRI, cardiomyocyte-specific ZBP1 deficiency ameliorates PANoptosis and reduces infarct size [62]. In isolated perfused guinea pig hearts, combined necrostatin-1 and Z-VAD-fmk reduces infarct size from 52% to 12%, whereas either monotherapy achieves only 33% [3]. Nanoparticles delivering PANoptosome-targeting combinations are capable of overcoming death pathway compensation. This capability marks them as a pivotal advancement in cardioprotective strategies. In experimental MIRI, nanoparticles simultaneously delivering cyclosporine A (mitochondrial pore inhibitor) and pitavastatin (HMG-CoA reductase inhibitor) to ischemic myocardium produce additive cardioprotection [137].

## 8. Conclusions and Future Perspectives

This review synthesizes evidence that LMP and the resulting cathepsin release constitute a proteolytic nexus for multi-modal cell death in MIRI. Cytosolic cathepsins, such as CTSB, cleave Bid to amplify mitochondrial apoptosis, activate NLRP3 to drive pyroptosis, and degrade ferritin to mobilize iron for ferroptosis. These proteolytic events converge on PANoptotic execution. The same mechanisms operate in endothelial cells. Cathepsin-dependent NLRP3 inflammasome activation and GSDMD-mediated pyroptosis disrupt microvascular barrier integrity and promote the no-reflow phenomenon. The interconnection of these pathways explains the limited efficacy of single-target cardioprotective strategies. Upstream modulation at the lysosomal-cathepsin axis may offer broader therapeutic benefit across both parenchymal and microvascular compartments.

### 8.1. Mechanistic Frontiers

The application of single-cell and spatial transcriptomics has illuminated the profound cellular heterogeneity in myocardial injury, particularly the dynamic reprogramming of cardiomyocytes, myeloid cells, and fibroblasts. Spatial neighborhood analysis has identified the endocardium as a portal for inflammatory cell recruitment [138]. Spatial multi-omics mapping links transcriptional programs to anatomical neighborhoods, providing a reference atlas for target discovery [139].

Cryo-electron microscopy has revealed the molecular machinery of inflammasome activation at near-atomic resolution. Structures of the active NLRP3 inflammasome disc show a decameric assembly in which ATP-bound NACHT domains interact centrally while PYD filaments recruit ASC [140]. The NLRP3 inhibitor MCC950 binds a cleft connecting NACHT subdomains [141]. Notably, the direct binding interface between CTSB and NLRP3 has not yet been resolved at atomic resolution. Structural characterization would enable orthosteric inhibitors that disrupt CTSB-mediated NLRP3 licensing without interfering with physiological lysosomal proteolysis.

Computational structure prediction offers a complementary route to the CTSB-NLRP3 interface. AlphaFold 2 and AlphaFold 3 predict protein structures and complexes with accuracy approaching experimental methods, enabling rapid modeling of the binding interface [142,143]. Integration with molecular dynamics simulations may accelerate the design of conformation-specific inhibitors. Such compounds could selectively distinguish between pathological cytosolic and physiological lysosomal protease states.

Ferroptosis depletes glutathione and cysteine while accumulating lipid peroxidation products such as 15-HpETE [39,74]. This metabolic fingerprint reflects organelle-specific iron trafficking. Lysosomal ferritinophagy and CTSB-mediated ferritin degradation release free iron. Lipid droplet remodeling changes PUFA-phospholipid substrate availability. Peroxisomal redox signaling provides parallel modulation. These organelle-specific inputs converge to determine ferroptotic sensitivity. Their integration into multi-omics frameworks will yield predictive models linking metabolic states to cell death execution in ischemic myocardium.

### 8.2. Biomarker-Guided Precision Cardioprotection

The translation of mechanistic insights into clinical benefit requires a paradigm shift in trial design. The CANTOS trial established that biomarker-guided patient selection, specifically targeting individuals with elevated high-sensitivity C-reactive protein, enabled canakinumab to reduce cardiovascular events where broad anti-inflammatory strategies had failed [113,123]. The recent CLEAR SYNERGY trial demonstrated that low-dose colchicine failed to reduce hard cardiovascular endpoints [128]. An additional consideration is that colchicine acts primarily through microtubule depolymerization rather than selective NLRP3 inhibition, and its pleiotropic actions may dilute any pathway-specific benefit. Neutral results in broad acute-MI populations should therefore not be read as failure of the NLRP3 axis itself. These findings underscore that anti-inflammatory interventions may require precise patient stratification based on factors beyond clinical syndromes alone.

Prospective cohort studies validate cell death pathway biomarkers in STEMI. Serum IL-1β predicts the no-reflow phenomenon with an AUC of 0.745 [112]. Elevated GSDMD within 48 h after PCI correlates with microvascular obstruction and intramyocardial hemorrhage [104]. Transcriptomic and metabolomic integration identifies ferroptosis-related signatures that stratify STEMI patients into distinct risk groups for major adverse cardiovascular events [144]. In selected cohorts, circulating NLRP3 inflammasome components discriminate ST-elevation from non-ST-elevation myocardial infarction with accuracy matching or exceeding conventional cardiac biomarkers [145]. These observations indicate that dynamic, multi-modal biomarker panels can support real-time cardiac risk stratification.

**Figure 2:**
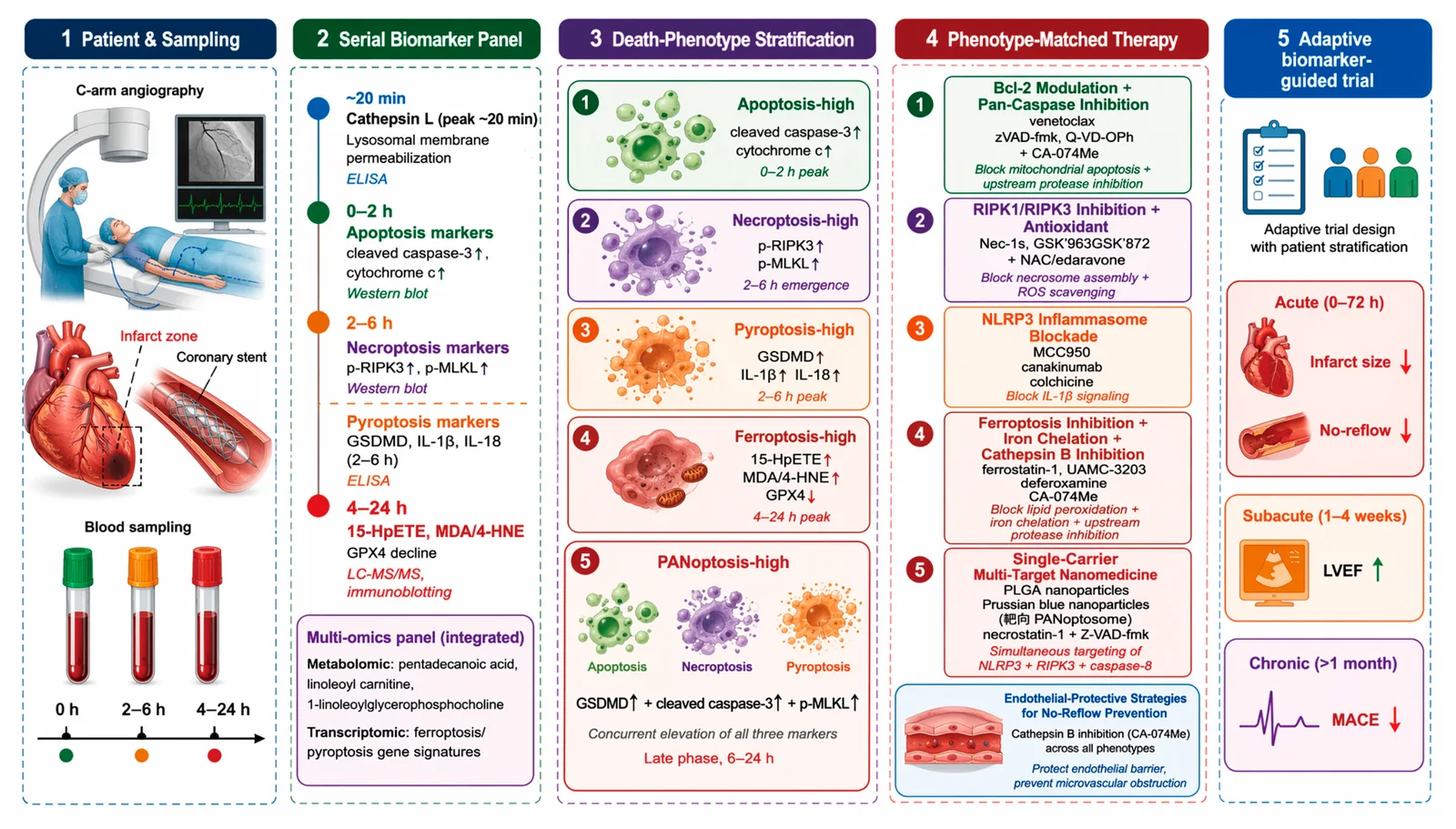
Biomarker-guided precision cardioprotection in myocardial ischemia–reperfusion injury. Stage 1, serial blood sampling after primary PCI. Stage 2, temporal biomarker panel tracking successive waves of death pathway activation from LMP through ferroptosis. Stage 3, stratification into five death-dominant phenotypes (apoptosis-high, necroptosis-high, pyroptosis-high, ferroptosis-high, and PANoptosis-high). Stage 4, phenotype-matched adjunctive therapies targeting specific death modalities or upstream convergence nodes. Stage 5, hierarchical endpoint assessment (acute, subacute, and chronic). The phenotype-stratified treatment scheme is hypothesis-generating and has not been validated in clinical trials. Endothelial-protective strategies are applied across all phenotypes. Fer-1, ferrostatin-1; DFO, deferoxamine; LVEF, left ventricular ejection fraction; MACE, major adverse cardiovascular events; MDA, malondialdehyde; 4-HNE, 4-hydroxynonenal; PCI, percutaneous coronary intervention; STEMI, ST-segment elevation myocardial infarction.

Current ESC and AHA/ACC guidelines emphasize timely reperfusion and secondary prevention yet do not incorporate biomarker-guided modulation of specific cell death pathways. Future guideline iterations may need to integrate precision medicine frameworks that align therapeutic decisions with individual cell death pathway activation patterns in the cardiac context (Figure 2). We emphasize that the phenotype-matched strategy illustrated in Figure 2 is a hypothesis-generating framework intended to guide mechanism-matched trial design. It has not been validated in clinical trials, and its clinical utility must be tested in prospective, mechanism-stratified studies.

### 8.3. Emerging Technologies

Human cardiac organoids derived from induced pluripotent stem cells recapitulate key features of myocardial injury in a human genetic context. Multi-cellular organoids containing cardiomyocytes, endothelial cells, and fibroblasts respond to hypoxic stress with apoptosis and calcium handling abnormalities [146,147]. These platforms enable high-throughput screening of cathepsin inhibitors and multi-modal cell death modulators, reducing the species gap from rodent models.

Advanced imaging technologies enable real-time visualization of pathological processes in vivo. Photoacoustic imaging visualizes CTSB activity in atherosclerotic plaques with tissue penetration exceeding 1.0 cm [106]. MRI-based iron mapping quantifies iron accumulation in IMI, distinguishing injured from remote tissue [148]. Furthermore, multimodal imaging can track ferroptosis and pyroptosis in the peri-infarct zone to guide targeted therapies.

### 8.4. Concluding Remarks

Several limitations qualify the conclusions drawn in this review. Rodent models differ from human cardiac physiology in heart rate, coronary anatomy, and collateral circulation. Young, healthy animals without comorbidities differ from aged, multimorbid patients. Driven by the intrinsic attenuation of cardioprotective signaling in diseased and comorbid hearts, primordial non-responsiveness blunts the efficacy of conditioning strategies in humans [149]. The cyclosporine A trials illustrate this gap. Robust preclinical efficacy did not translate into clinical benefits in large STEMI cohorts. This failure is largely attributed to concurrent medications, variable ischemia duration, and high comorbidity burden [130,131]. Translation is further complicated by species differences in cardiac physiology, infarct size scaling, and inflammatory kinetics. These issues are compounded by the comorbidities and background medications of patients, which healthy young laboratory animals do not carry. Furthermore, the diseased human myocardium is often non-responsive to conditioning interventions [149]. This is evidenced by the neutral outcomes of the CIRCUS and CYCLE trials. Together, these differences help explain why robust preclinical cardioprotection, including cathepsin-directed strategies, may not translate into clinical benefit. Multi-cellular cardiac organoids may bridge this species divide.

Therapeutic time windows are narrow and ill-defined. Serum CTSL rises immediately upon primary PCI reflow and correlates with infarct severity [46]. The critical proteolytic window opens within minutes of reperfusion. The CIRCUS and CYCLE trials demonstrated that pre-procedural cyclosporine A failed to reduce infarct size. This failure highlights the challenge of synchronizing pharmacological delivery with emergent reperfusion [130,131]. Specificity remains a challenge. CTSS maintains essential roles in invariant chain processing and antigen presentation, while CTSK is required for physiological bone remodeling [119,150]. CTSK inhibition by odanacatib increased cardiovascular risk in osteoporosis trials, terminating its development [119]. Systemic inhibition therefore disrupts these physiological processes. Conformation-specific inhibitors that exploit distinct proteolytic preferences at pathological versus lysosomal pH [18], or localized delivery systems restricting drug exposure to ischemic tissue, may resolve this tension [135].

Compensatory activation limits single-target efficacy. Suppressing apoptosis may shift cells toward ferroptosis or necroptosis. Inhibiting ferroptosis may amplify pyroptosis through inflammasome activation. Combined necroptosis and apoptosis blockade reduced infarct size from 52% to 12% in isolated perfused hearts [3]. Monotherapy achieved only modest protection. Multi-target combination therapy or upstream inhibition at convergence nodes such as LMP is required.

Long-term safety of multi-target combinations is unknown. The NLRP3 inhibitor MCC950, despite potent preclinical efficacy, was discontinued in Phase II rheumatoid arthritis trials owing to hepatotoxicity mediated by off-target inhibition of carbonic anhydrase 2 [132,133]. Simultaneous inhibition of multiple death modalities may further impair physiological cell turnover, immune surveillance, and tissue repair. Addressing these limitations will require convergent advances in human-relevant experimental systems, analytically validated biomarkers, and trial designs that stratify patients by mechanistic pathway activation.

Biological sex is an additional and understudied modifier of MIRI. In most rodent I/R models, females sustain smaller infarcts and show better functional recovery than males. This advantage is most pronounced before reproductive senescence and is attributed to estrogen-receptor signaling. It is also driven by sex-dependent mitochondrial protein phosphorylation, such as ALDH2 and α-ketoglutarate dehydrogenase, which limits ROS generation during reperfusion [151,152,153]. Whether sex influences lysosomal stability or cathepsin release remains unknown. No MIRI study has directly compared LMP magnitude, cathepsin release, or cathepsin-inhibitor efficacy between sexes. Furthermore, many of the preclinical studies cited in this review used male-only cohorts. Sex-balanced experimental designs and sex-disaggregated biomarker analyses should therefore be a priority for future cathepsin-oriented MIRI research.

Future clinical trials should adopt biomarker-guided enrichment to identify patients with predominant ferroptotic or pyroptotic signatures, the subgroup most likely to benefit from pathway-matched adjunctive therapy. The lysosomal-cathepsin axis remains a compelling therapeutic target. Its clinical pursuit demands mechanistic rigor, biomarker-guided patient selection, and careful consideration of the physiological roles of these proteases beyond the injured myocardium and coronary microcirculation. Understanding the temporal dynamics and predominant influence of individual death mechanisms is crucial for precisely targeting therapeutic interventions.

## Figures and Tables

**Table 1:** Comparative features of programmed cell death modalities in myocardial ischemia–reperfusion injury.

Modality	Morphological Features	Key Executioners	Cathepsin-Mediated Mechanism
Apoptosis	Cell shrinkage, membrane blebbing, nuclear fragmentation	Caspase-3/7, Caspase-9, Cytochrome c	CTSB/CTSD cleave Bid → tBid → MOMP → caspase-9/3 [25,42]
Pyroptosis	Membrane pore formation, cell swelling, IL-1β/IL-18 release	GSDMD, Caspase-1, NLRP3, ASC	CTSB binds NLRP3 → inflammasome → caspase-1 → GSDMD [27]
Ferroptosis	Iron-dependent lipid peroxidation, no caspase activation	GPX4, ALOX15, NCOA4	CTSB may degrade ferritin → Fe^2+^ → lipid peroxidation (non-cardiac evidence; limited cardiac proof) [44,45]
Necroptosis	Membrane pore, cell lysis, DAMP release	RIPK1, RIPK3, MLKL	Indirect. LMP may amplify signaling; direct CTSB links undefined in cardiac cells [40,41,80]
PANoptosis	Integrated execution of pyroptosis + apoptosis + necroptosis via PANoptosome platforms	NLRP3, ASC, caspase-1/8, RIPK3, MLKL, GSDMD	Inferred through NLRP3 activation and Bid cleavage; canonical cardiomyocyte PANoptosome not directly shown [12,27,42,62]

NOTE: Timing reflects experimental MIRI models and clinical observations; individual variation exists. CTSB, cathepsin B; CTSD, cathepsin D; LMP, lysosomal membrane permeabilization; MOMP, mitochondrial outer membrane permeabilization; GSDMD, gasdermin D; ASC, apoptosis-associated speck-like protein; GPX4, glutathione peroxidase 4; ALOX15, 15-lipoxygenase; NCOA4, nuclear receptor coactivator 4; RIPK, receptor-interacting protein kinase; MLKL, mixed lineage kinase domain-like. Bracketed numbers refer to the reference list of the accompanying manuscript.

**Table 2:** Experimental models used to study cathepsins and regulated cell death in myocardial ischemia–reperfusion.

Model and Representative Protocol	Principal Strengths	Key Limitations	Interpretive Caveats for Cathepsin–Cell Death Studies
Isolated cardiomyocyte hypoxia/reoxygenation (neonatal rat, adult rodent, or hiPSC-derived cardiomyocytes; simulated ischemia 1–24 h, reoxygenation 1–24 h)	Cell-autonomous mechanism; easy genetic and pharmacological manipulation; high throughput; human relevance via hiPSC-CMs	No flow, endothelium, blood cells, or neurohormonal input; neonatal and hiPSC cardiomyocytes are metabolically and structurally immature	Reports cardiomyocyte-intrinsic LMP and death execution directly; cannot capture leukocyte-driven amplification or microvascular events
Langendorff-perfused isolated heart (retrograde crystalloid perfusion; global ischemia 20–40 min, reperfusion 2 h)	Intact myocardial architecture with contractile readout; precise control of perfusate composition and timing; reproducible infarct sizing	Acellular perfusate lacks neutrophils, monocytes, complement, and platelets; short reperfusion window	Underestimates inflammation-driven pyroptosis and late-phase cathepsin release; protection observed here may not translate in vivo
Rodent in vivo coronary (LAD) occlusion/reperfusion (ischemia 30–60 min; reperfusion 2 h–7 days)	Full systemic context: circulating inflammatory cells, complement, neurohormonal signaling; chronic endpoints (remodeling, fibrosis)	Species differences in heart rate, collateral flow, and cathepsin isoform expression; surgical and biological variability	The only small-animal setting in which leukocyte-derived cathepsins (e.g., macrophage CTSS) are visible; the death-modality mix depends strongly on ischemia duration
Large-animal, particularly porcine closed-chest, I/R (balloon occlusion 40–90 min; reperfusion days to weeks)	Human-like cardiac anatomy, size, and hemodynamics; imaging- and catheter-based translational readouts	High cost and low throughput; limited genetic tools; few cathepsin-specific studies to date	Best predictor of clinical efficacy of cathepsin inhibitors; currently an evidence gap—most cathepsin data remain rodent-derived
Human tissue and clinical sampling (autopsy/explant histology; plasma cathepsin activity during STEMI and PCI)	Direct clinical relevance; biomarker potential (e.g., circulating CTSB activity, plasma RIPK3 [14])	Observational and confounded by comorbidities and concomitant medication; tissue largely restricted to end-stage disease	Establishes association rather than mechanism; the sampling window determines which death wave is captured

NOTE: hiPSC-CM, human induced pluripotent stem cell-derived cardiomyocyte; LAD, left anterior descending coronary artery; I/R, ischemia/reperfusion; LMP, lysosomal membrane permeabilization; CTSS, cathepsin S; CTSB, cathepsin B; RIPK3, receptor-interacting protein kinase 3; NOX2, NADPH oxidase 2; NLRP3, NOD-, LRR- and pyrin domain-containing protein 3; STEMI, ST-segment-elevation myocardial infarction; PCI, percutaneous coronary intervention.

**Table 3:** Circulating biomarkers of cathepsin activity and multi-modal cell death in myocardial ischemia–reperfusion injury.

Biomarker	Associated Modality	Detection Modality	Clinical Platform Readiness	Peak Time Post-Reperfusion	Key Evidence	Prognostic Value
Serum Cathepsin L	Multi-modal (LMP, proteolytic burden)	ELISA, immunoturbidimetry	Tier (ii). Clinical-grade (available in clinical chemistry labs)	Immediate (<20 min) to 24 h	Peaks in coronary sinus within minutes of reperfusion; correlates with infarct size by CMR at 24 h and predicts LVEF at 6 months [46]	Dynamic indicator of lysosomal injury severity; early stratification marker
Serum Cathepsin S	Atherosclerosis, plaque vulnerability	ELISA	Tier (ii). Clinical-grade (research use)	Chronic plaque elevation (not acute phase-specific)	Elevated levels associated with increased cardiovascular mortality in population cohorts [100]	Risk stratification in chronic coronary disease; less specific for acute MI/RI
Serum Cystatin C	Endogenous cathepsin inhibition; GFR	Immunoturbidimetry, ELISA	Tier (i). Clinical-grade (widely available)	Acute phase + serial monitoring	Rises acutely in STEMI reflecting protease-inhibitor imbalance; HR 3.2 for 12-month mortality in acute HF when above median; predicts MACE in chronic HF independent of traditional risk factors and BNP [102,103]	Dual biomarker: reflects both glomerular filtration and cathepsin inhibitory capacity; readily deployable
GSDMD (full-length and N-terminal fragment)	Pyroptosis execution	ELISA (commercially available)	Tier (ii). ELISA platforms validated; single-cohort clinical validation	2–6 h post-PCI	Significantly elevated after PCI in STEMI vs. pre-procedure and healthy controls; independently associated with MVO, IMH, and larger myocardial injury [104]	Prognostic for microvascular obstruction and infarct severity; potential for real-time injury assessment
IL-1β/IL-18	Pyroptosis (NLRP3 inflammasome activation)	ELISA, high-sensitivity assays	Tier (ii). Clinical-grade (hs-IL-1β in development)	2–6 h post-reperfusion	Matured through caspase-1 cleavage; detectable within hours of ACS onset; IL-1β independently predicts no-reflow (AUC 0.745) [112]; CANTOS validated IL-1β suppression as therapeutic target [113]	Established inflammatory mediator; CANTOS validated IL-1β suppression as therapeutic target [113]
Circulating ASC specks	Pyroptosis (inflammasome structural marker)	ELISA (specialized)	Tier (iii). Research-grade (standardized assay lacking)	2–12 h post-reperfusion	Oligomerized ASC protein released from activated inflammasomes; structural biomarker [108]	May reflect cumulative inflammasome burden; requires analytical validation
15-HpETE (ALOX15-derived)	Ferroptosis priming signal	LC-MS/MS (targeted metabolomics)	Tier (iii). Research-grade (requires mass spectrometry; low throughput for ED)	4–24 h post-reperfusion	Specific ferroptosis-associated metabolite identified in MIRI; accumulates during ischemia and drives ferroptosis upon reperfusion [39]	Mechanistically specific for ferroptosis; limited by platform availability and turnaround time
MDA/lipid peroxidation products	Ferroptosis (lipid peroxidation)	LC-MS/MS, colorimetric assays	Tier (iii). Research-grade (colorimetric)/Clinical-grade (LC-MS/MS in specialized centers)	4–24 h post-reperfusion	Metabolomic profiling in STEMI patients undergoing primary PCI identifies plasma metabolite panels (pentadecanoic acid, linoleoyl carnitine, 1-linoleoylglycero-phosphocholine) that discriminate infarct size with >80% predictive accuracy [114]	Reflects oxidative stress and lipid peroxidation burden; metabolite panels offer superior specificity over single products
GPX4 (serum/plasma levels)	Ferroptosis (antioxidant capacity)	ELISA, Western blot	Tier (iii). Research-grade (no standardized serum assay)	Progressive decline during sustained reperfusion	Quantitative proteomics shows decline during ischemic injury; pharmacological inhibition or genetic ablation exacerbates ferroptosis [38]	Functional biomarker of ferroptotic execution; dynamic monitoring potential
Non-transferrin-bound iron (NTBI)	Ferroptosis (labile iron pool)	Colorimetric, HPLC	Tier (ii). Clinical-grade (available in iron metabolism panels)	Baseline + acute phase	Reflects iron available to catalyze lipid peroxidation; elevated in iron overload states	Complementary to ferritin; may guide iron chelation therapy selection
p-RIPK3/p-MLKL	Necroptosis execution	Western blot (tissue); ELISA (serum, in development)	Tier (iii). Research-grade (tissue validated; serum assay lacking)	4–24 h post-reperfusion	Phosphorylated MLKL is the definitive executioner marker; elevated in ischemic myocardium; kinase inhibition reduces serum CK-MB and troponin [109,110]	Definitive necroptosis marker; serum assay development is priority for clinical translation
Cleaved Caspase-3/Caspase-8	Apoptosis execution	ELISA, Western blot	Tier (iii). Research-grade (specialized labs)	0–4 h post-reperfusion (early)	Classical apoptosis executioners; elevated in serum and tissue during early reperfusion	Less specific for MI/RI (elevated in other apoptotic conditions); kinetic profile may distinguish ischemic from non-ischemic injury
PANoptosis composite panel (p-MLKL, GSDMD-NT + Cleaved Caspase-3)	PANoptosis (integrated execution)	Multi-platform (ELISA + WB + MS)	Tier (iii). Research/developmental; no validated clinical assay	Variable (component-specific)	Co-detection of three executioner markers supports PANoptosis-like activation; multi-omics models achieve AUC 0.874 in training and 0.756–0.780 in validation [115]	Future precision medicine tool; requires prospective validation in STEMI cohorts

NOTE: LMP, lysosomal membrane permeabilization; GFR, glomerular filtration rate; CMR, cardiac magnetic resonance; LVEF, left ventricular ejection fraction; PCI, percutaneous coronary intervention; STEMI, ST-segment elevation myocardial infarction; MVO, microvascular obstruction; IMH, intramyocardial hemorrhage; ACS, acute coronary syndrome; AUC, area under the curve; 15-HpETE, 15-hydroperoxyeicosatetraenoic acid; LC-MS/MS, liquid chromatography-tandem mass spectrometry; ED, emergency department; MDA, malondialdehyde; WB, Western blot; MS, mass spectrometry; HR, hazard ratio; HF, heart failure; MACE, major adverse cardiovascular events; BNP, B-type natriuretic peptide. Bracketed numbers refer to the reference list of the accompanying manuscript.

**Table 4:** Clinical trials of anti-inflammatory and cell death-modulating therapies in cardiovascular disease.

Trial/Agent	Phase	Target/Mechanism	Population	Primary Endpoint	Key Result	Implications for Cathepsin-Mediated Therapy	Development Status
CANTOS (Canakinumab)	III	IL-1β neutralization (Downstream of NLRP3 inflammasome)	10,061 patients with prior MI and hs-CRP ≥ 2 mg/L	MACE (CV death, non-fatal MI, non-fatal stroke)	15% RRR (HR 0.85, 95% CI 0.74–0.98); IL-1β-driven inflammation causally linked to atherothrombosis [113]	Proof-of-concept that IL-1β-driven inflammation reduces CV events; indirect support for NLRP3-pyroptosis axis	Marketed (periodic fever syndromes); cardiovascular indication not pursued
LoDoCo2 (Colchicine 0.5 mg/d)	III	NLRP3 inflammasome assembly blockade; microtubule inhibition	5522 patients with chronic coronary disease	Composite of CV death, MI, ischemic stroke, or ischemia-driven coronary revascularization	31% RRR vs. placebo over a median follow-up of 28.6 months; effective in chronic setting [118]	Orally available NLRP3 anti-inflammatory strategy feasible; however, acute MI/RI context (CLEAR SYNERGY) showed neutral results [128]	Generic; low-dose colchicine approved for CV risk reduction (US, 2023)
COLCOT (Colchicine 0.5 mg/d)	II	NLRP3 inflammasome inhibition	4745 patients within 30 days of MI	Composite of CV death, resuscitated cardiac arrest, MI, stroke, or urgent coronary revascularization	23% RRR over a median follow-up of 22.6 months; benefit driven by reduction in urgent coronary revascularization [117]	Early post-MI anti-inflammatory intervention may have time-limited benefit; narrow therapeutic window	Generic (same agent as LoDoCo2)
CLEAR SYNERGY (Colchicine 0.5 mg/d)	III	NLRP3 inflammasome inhibition	7062 patients with acute MI undergoing PCI	Composite of CV death, recurrent MI, stroke, or unplanned revascularization over 3 years	Neutral; colchicine reduced CRP but not hard clinical endpoints [128]	Highlights that anti-inflammatory monotherapy in broad acute MI populations may be insufficient; biomarker-guided enrichment may be needed	Generic
CIRT (Low-dose methotrexate)	Large randomized outcome trial	Broad anti-inflammatory (Folate antagonist)	4786 patients with prior MI or multivessel CAD and diabetes/metabolic syndrome	Composite of CV death, non-fatal MI, non-fatal stroke, or unstable angina requiring revascularization	Neutral; no reduction in IL-1β, IL-6, or CRP [129]	Non-specific anti-inflammatory approach without inflammasome targeting ineffective; supports pathway-specific inhibition	Generic
CIRCUS (Cyclosporine A)	II/III	MPTP inhibition (Mitochondrial protection)	970 patients with STEMI undergoing primary PCI	Infarct size (CMR at 5 days) and clinical endpoints	Neutral; no reduction in infarct size or clinical events [130]	Demonstrates primordial non-responsiveness; preclinical efficacy in healthy young animals did not translate to comorbid human STEMI	Marketed (immunosuppression); cardioprotection development halted
CYCLE (Cyclosporine A)	II	MPTP inhibition	410 patients with STEMI undergoing primary PCI	ST-segment resolution (electrocardiographic surrogate)	Neutral; no significant improvement in ST-segment resolution [131]	Reinforces narrow therapeutic window and species gap in cardioprotective translation	Marketed (immunosuppression)
Dapansutrile (OLT1177)	IB	Selective oral NLRP3 inhibitor	Patients with systolic heart failure	Safety and tolerability (exploratory efficacy)	Safe and well tolerated, with preliminary efficacy signals; the most clinically advanced selective NLRP3 inhibitor relevant to cardiovascular indications [125]	Demonstrates clinical feasibility of oral NLRP3 inhibition in cardiovascular patients	Phase IB completed (heart failure); Phase II programs in non-cardiac indications
Odanacatib	III program (osteoporosis)	Selective cathepsin K inhibition	Postmenopausal women with osteoporosis	Fracture endpoints with adjudicated cardiovascular safety	Cardiovascular safety signal (increased stroke risk) observed; development terminated [119]	Underscores that systemic cathepsin inhibition requires dedicated cardiovascular safety monitoring	Development discontinued (osteoporosis program)
MCC950 (selective NLRP3 inhibitor)	Preclinical (porcine); human Phase II discontinued	Selective NLRP3 inflammasome inhibition	Pig model of acute MI (LAD occlusion/reperfusion)	Infarct size, LVEF, and hemodynamic parameters	Reduced infarct size and preserved cardiac function; validated in large-animal MI/RI [37]	Supports translatability of NLRP3 inhibition to large-animal physiology; Phase II development discontinued due to hepatotoxicity (off-target CA2 inhibition) [132,133]	Phase II discontinued (hepatotoxicity); remains a preclinical tool compound
CA-074Me (CTSB inhibitor)	Preclinical; Clinical pharmacokinetics pending	Selective cathepsin B inhibition	Mouse model of MI/RI (LAD ligation/reperfusion)	Infarct size, serum cTnI, CK-MB	Attenuated infarct size and reduced cardiac injury biomarkers; inhibited caspase-3 activation [36]	Direct evidence that cathepsin B inhibition protects against MI/RI; clinical translation requires human-relevant pharmacokinetics and safety profiling	Preclinical tool compound

NOTE: IL, interleukin; hs-CRP, high-sensitivity C-reactive protein; MACE, major adverse cardiovascular events; RRR, relative risk reduction; HR, hazard ratio; CI, confidence interval; CAD, coronary artery disease; MPTP, mitochondrial permeability transition pore; CMR, cardiac magnetic resonance; LAD, left anterior descending artery; LVEF, left ventricular ejection fraction; cTnI, cardiac troponin I; CK-MB, creatine kinase-MB; CTSB, cathepsin B; CA2, carbonic anhydrase 2. Evidence tiers: (i) preclinical MIRI studies (MCC950, CA-074Me); (ii) clinical trials in MIRI or acute coronary syndromes (CIRCUS, CYCLE, CLEAR SYNERGY, COLCOT); (iii) clinical trials in other cardiovascular or non-cardiovascular indications (CANTOS, LoDoCo2, CIRT, dapansutrile, odanacatib). Bracketed numbers refer to the reference list of the revised manuscript.

## Data Availability

No new data were created or analyzed in this study. Data sharing is not applicable to this article.
